# Mitochondrial Effects of Hydromethylthionine, Rivastigmine and Memantine in Tau-Transgenic Mice

**DOI:** 10.3390/ijms241310810

**Published:** 2023-06-28

**Authors:** Constantin Kondak, Michael Leith, Thomas C. Baddeley, Renato X. Santos, Charles R. Harrington, Claude M. Wischik, Gernot Riedel, Jochen Klein

**Affiliations:** 1 Institute of Medical Sciences, University of Aberdeen, Foresterhill, Aberdeen AB25 2ZD, UK; 2 Institute of Pharmacology and Clinical Pharmacy, Goethe University Frankfurt, Max-von-Laue-Str. 9, 60438 Frankfurt, Germany; 3 Department of Chemistry, School of Natural and Computing Sciences, University of Aberdeen, Aberdeen AB24 3UE, UK; 4 TauRx Therapeutics Ltd., Aberdeen AB24 5RP, UK

**Keywords:** microdialysis, Alzheimer’s disease, rivastigmine, memantine, complex I, cytochrome c oxidase

## Abstract

Tau protein aggregations are important contributors to the etiology of Alzheimer’s disease (AD). Hydromethylthionine (HMT) is a potent inhibitor of tau aggregation in vitro and in vivo and is being developed as a possible anti-dementia medication. HMT was also shown to affect the cholinergic system and to interact with mitochondria. Here, we used tau-transgenic (L1 and L66) and wild-type NMRI mice that were treated with HMT, rivastigmine and memantine and with combinations thereof, for 2–4 weeks. We measured HMT concentrations in both brain homogenates and isolated mitochondria and concentrations of glucose, lactate and pyruvate in brain by microdialysis. In isolated brain mitochondria, we recorded oxygen consumption of mitochondrial complexes by respirometry. While rivastigmine and memantine lowered mitochondrial respiration, HMT did not affect respiration in wild-type animals and increased respiration in tau-transgenic L1 mice. Glucose and lactate levels were not affected by HMT administration. The presence of HMT in isolated mitochondria was established. In summary, traditional anti-dementia drugs impair mitochondrial function while HMT has no adverse effects on mitochondrial respiration in tau-transgenic mice. These results support the further development of HMT as an anti-dementia drug.

## 1. Introduction

Alzheimer’s disease (AD) is a substantial burden to individuals. Due to rising numbers of AD patients, it is also a major problem for health care systems. This makes the development of new therapeutic concepts for treating AD an important goal [1,2]. The drugs that are currently in use, e.g., acetylcholinesterase (AChE) inhibitors and memantine, act symptomatically and delay the disease progression. Unfortunately, potential disease-modifying drugs such as those that reduced Aβ peptides in the brain have not yielded positive outcomes in clinical studies [3]. Therefore, current drug development for AD focuses on aspects such as tau aggregation as well as energy metabolism and mitochondrial function in the brain [3,4]. Cerebral glucose consumption is decreased early on in AD, and this is reflected in reduced activities of glycolytic enzymes and enzymes of the tricarboxylic acid cycle [5,6]. Mitochondrial complex activities in AD patients are also impaired [7,8,9]. As transgenic mouse models of AD also display mitochondrial dysfunction [10,11,12], “brain energy rescue” has been suggested as a promising therapeutic approach for AD [4].

Intracellular tau aggregates are a prominent feature of AD and were used by Eva and Heiko Braak to stage the progression of neurodegeneration [13]. Starting in the entorhinal cortex, tau aggregation spreads to the hippocampus and neocortical regions and causes the accumulation of insoluble paired helical filaments (PHFs) [14,15]. Tau protein plays a crucial role in axonal transport, and its loss of function leads to neurodegeneration [15,16]. The accumulation of tau protein aggregates correlates with neuronal cell death in AD and is associated with mitochondrial pathology, including changes of fission and fusion processes and of mitophagy [9,17]. Dissolution or removal of tau aggregates may therefore be a promising way to reestablish mitochondrial function and more globally alleviate the progression of dementia. We reasoned that this might be achieved by the repeated administration of tau aggregation inhibitors.

Methylthioninium chloride (MTC, commonly known as methylene blue) was the first selective tau aggregation inhibitor identified in vitro [18]. MTC treatment was found to slow cognitive decline at a dose of 138 mg/day as monotherapy in a phase 2 trial in AD [19]. A reduced form of this compound, hydromethylthionine (HMT; the new International Non-Proprietary Name for leucomethylthioninium, LMT) was tested in two phase 3 trials in mild to moderate AD. Treatment in doses of 150–250 mg/day was compared with a low dose of 8 mg/day intended as an inactive control. There were no differences between high and low doses in either of the two trials [20,21]. The lack of dose-dependent difference has since been explained by the fact that, in contrast to MTC, 8 mg/day is the minimum effective dose of HMT and there is a response plateau at higher levels of exposure [22]. Significant differences were seen in both trials between patients receiving HMT as monotherapy and those receiving HMT as an add-on to standard symptomatic treatments [20,21].

We recently set out to mimic this dissociation in a preclinical trial in tau-transgenic mice. The interactions of HMT with rivastigmine and memantine surprisingly revealed that pretreatment with rivastigmine or memantine prevented the beneficial effect of HMT on the cholinergic system [23,24]. With this in mind, the present study investigated the effects of HMT, rivastigmine and memantine on brain energy metabolism including mitochondrial activity. We monitored glucose and lactate levels by microdialysis and measured mitochondrial respiration ex vivo in isolated brain mitochondria.

## 2. Results

### 2.1. Energy Metabolites

**Figure 1:**
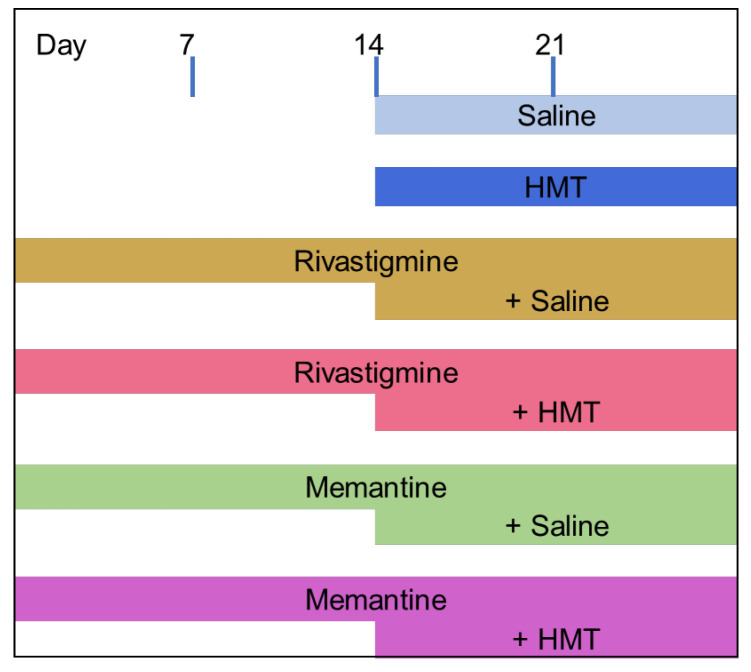
Administration regime for different drug treatments. Rivastigmine (0.5 mg/kg/d) and memantine (1 mg/kg/d) were administered using a subcutaneously placed osmotic mini-pump, which was implanted 28 days prior to sampling/tissue harvest. Saline (0.2 mL/kg/d) or HMT (5 mg/kg/d) were given daily by oral gavage from day 15 for two weeks before sampling and analysis. Probe implantation surgery was performed on day 26, followed by two consecutive days of microdialysis experiments. Brain tissue for measurement of mitochondrial respiration was obtained on day 28. For each cohort (for both microdialysis and mitochondrial respiration), ten animals were intended. The exact number of animals for each experiment is given in the respective figure description. Five animals were lost due to surgical complications, and a further four animals were lost due to blocked microdialysis probes. Furthermore, some data points were identified as outliers and were therefore excluded from analysis (see Section 2 or details).

We first determined the concentrations of energy metabolites in blood and brain from all groups (Figure 1).

**Figure 2:**
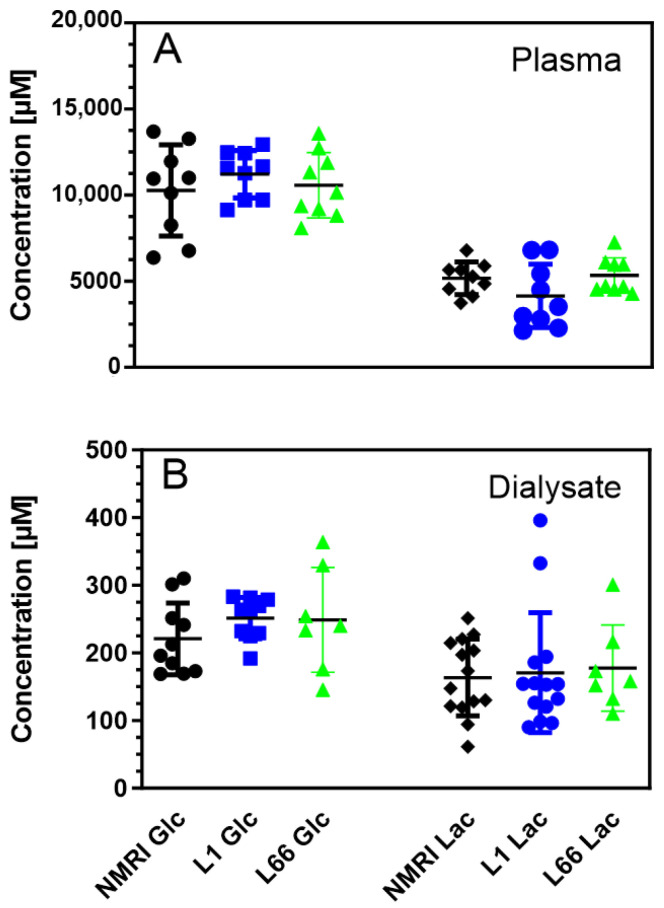
Concentrations of glucose (Glc) and lactate (Lac) in plasma (**A**) and microdialysate (**B**) in wild-type (NMRI), L1 and L66 mice. Data are presented as scatter plot with mean and standard deviation; variable numbers are due to the inclusion of two cohorts of mice (N = 9 for plasma, N = 7–14 for microdialysates; see Section 2). For both parameters, two-way ANOVA with genotype as between subject and metabolite as within-subject factors revealed a main effect of metabolite (Glc vs. Lac; F values > 26; *p* values < 0.0001) but not for terms including phenotype.

Basal glucose levels in blood plasma of saline-treated NMRI mice were 10.9 ± 0.9 mM, and basal lactate levels were 5.2 ± 0.3 mM (N = 9; Figure 2A). In the microdialysates, glucose levels were 208 ± 21 µM, and lactate levels were 148 ± 15 µM (N = 9; Figure 2B). The values for the L1 and L66 mice were not significantly different from wild-type NMRI mice (Figure 2).

**Figure 3:**
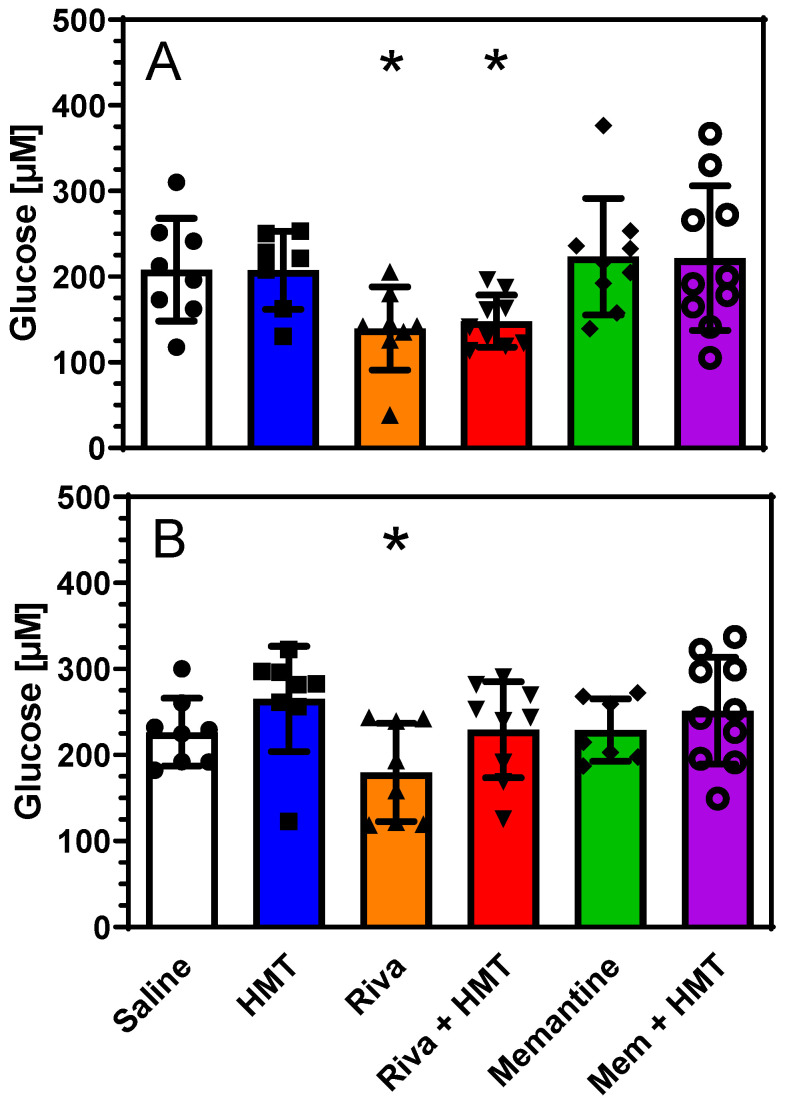
Glucose levels in microdialysates of (**A**) wild-type mice (NMRI: Saline n = 8; HMT n = 7; Rivastigmine (Riva) n = 8; Riva + HMT n = 9; Memantine (Mem) n = 9; Mem + HMT n = 10) and (**B**) L1 mice (Saline n = 8; HMT n = 8; Riva n = 8; Riva + HMT n = 9; Memantine n = 7; Mem + HMT n = 10) after administration of rivastigmine, memantine and/or HMT as shown in Figure 1. Data are presented as scatter plots and bars represent mean ± SD. One-way ANOVA: (A) F_5,50_ = 3.37; *p* = 0.012. (**B**) F_5,49_ = 2.41; *p* < 0.05. Asterisks indicate significant *t*-test comparison with control (saline) administration; * *p* < 0.05. Six data points in different groups of mice with different genetic backgrounds were identified as outliers and were excluded.

Interestingly, significant changes were observed after drug exposure. Treatment with rivastigmine decreased glucose levels in microdialysates by 33% in NMRI and by 21% in L1 mice (*p* < 0.05). This effect was prevented by concomitant treatment with HMT in tau-transgenic L1 but not in wild-type NMRI mice (Figure 3). Rivastigmine also increased the lactate/pyruvate ratio in NMRI and L1 mice, an effect that was antagonized by HMT in both strains. This combination of effects suggested an inhibition of mitochondrial respiration and aerobic glycolysis by rivastigmine, while administration of either HMT or memantine did not affect energy metabolites (Figure 3).

### 2.2. Mitochondrial Respiration

**Figure 4:**
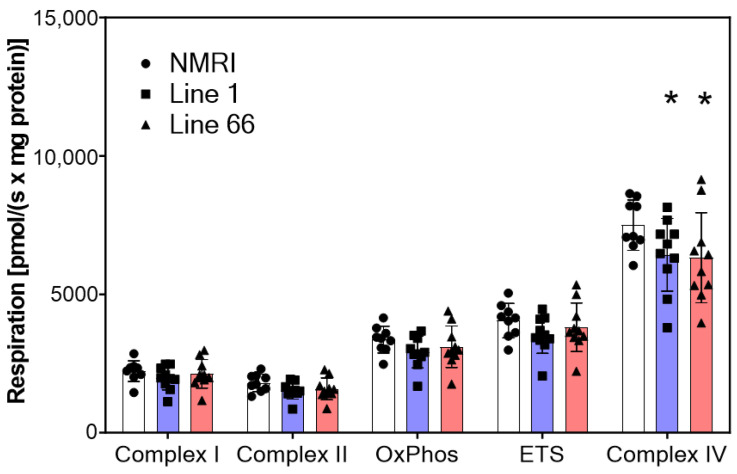
Oxygen flux of mitochondrial complexes in NMRI, Line 1 and Line 66 mice. Substrates, uncouplers and inhibitors were used to measure complex activities in isolated brain mitochondria as described in Section 2. Raw data were normalized to mitochondrial citrate synthase activity. Data are shown as scatter plots and mean ± SD for 10 animals in each condition. Statistical analysis between genotypes included a two-way ANOVA with protein complex as repeated measures and revealed a significant main effect of protein complex and a genotype by protein complex interaction (F values > 2.6; *p* values < 0.012). Asterisks indicate significant lowering of levels compared with NMRI controls (*t*-test: * *p* < 0.05). Data from one NMRI mouse were removed (outlier) since they were too low throughout all measurements (<30% of mean).

The basal activity of mitochondrial complexes for the three mouse strains is shown in Figure 4. Complex I and II activities were similar in all three strains, leading to a respiration of approximately 3200 pmol/(s × mg) for oxidative phosphorylation (OxPhos). The oxygen consumption of the OxPhos reached 82% of the maximum ETS which suggests efficient ATP synthesis from the transported electrons. Complex IV activity was considerably higher than ETS; however, this value was obtained under artificial conditions of substrate (and electron) supply with ascorbic acid and TMPD (see Section 2). While the overall two-way ANOVA confirmed a main difference between readouts for the mitochondrial complexes and an interaction with genotype (F’s > 2.6; *p* < 0.012), a planned comparison between NMRI and L1 cohorts returned a strong trend towards a deficit between genotypes (F_1,17_ = 3.85; *p* = 0.066). It must be noted that L1 and L66 mice had significantly less complex IV activities than wild-type mice (Figure 4).

**Figure 5:**
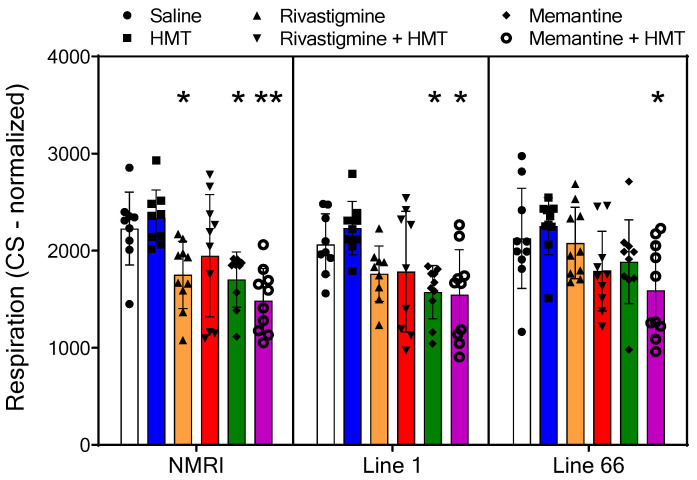
Oxygen flux of *Complex I* in NMRI, Line 1 and Line 66 mice with different treatment conditions. Substrates, uncoupler and inhibitors were used to measure complex I (see Section 2 for details). Raw data were normalized to mitochondrial citrate synthase activity and are presented as scatter plots with means ± SD of 10 experiments for each group. In total, six measurements were deemed to be outliers due to very low values (below 25% of mean in respective groups: n = 1 for each treatment condition: NMRI—saline, HMT; L1—saline, HMT, Riva, Riva + HMT. An overall two-way ANOVA only returned a main effect of treatment (F_5,155_ = 12.7; *p* < 0.0001), but not for the term genotype (F values < 1.5). Consequently, genotypes were individually probed by one-way ANOVA followed by Bonferroni’s multiple comparison test (asterisks * *p* < 0.05; ** *p* < 0.01).

**Figure 6:**
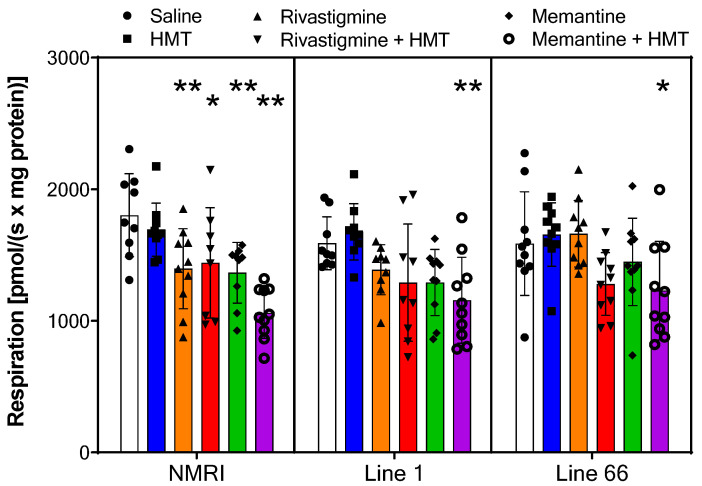
Oxygen flux of *Complex II* in NMRI, Line 1 and Line 66 mice with different treatment conditions. Substrates, uncoupler and inhibitors were used to measure complex II as described in Section 2. Raw data were normalized to mitochondrial citrate synthase activity and are presented as scatter plots and means ± SD for 10 subjects in each condition. Outliers (extremely high/low) were identified in the following groups with n = 1: NMRI—saline, memantine; L1—saline, HMT, Rivastigmine, Rivastigmine + HMT; n = 2: NMRI Rivastigmine + HMT). As for complex I above, the two-way ANOVA only revealed significant differences between the level of complex II activation between treatment groups (F_5,153_ = 14; *p* < 0.0001), but not for the term genotype (F values < 1.55). The genotype-specific ANOVA with Bonferroni post hoc confirmed a lowering of complex II levels in selected treatment groups (see asterisks for comparison with saline control: * *p* < 0.05; ** *p* < 0.01), most consistently when memantine was combined with HMT.

**Figure 7:**
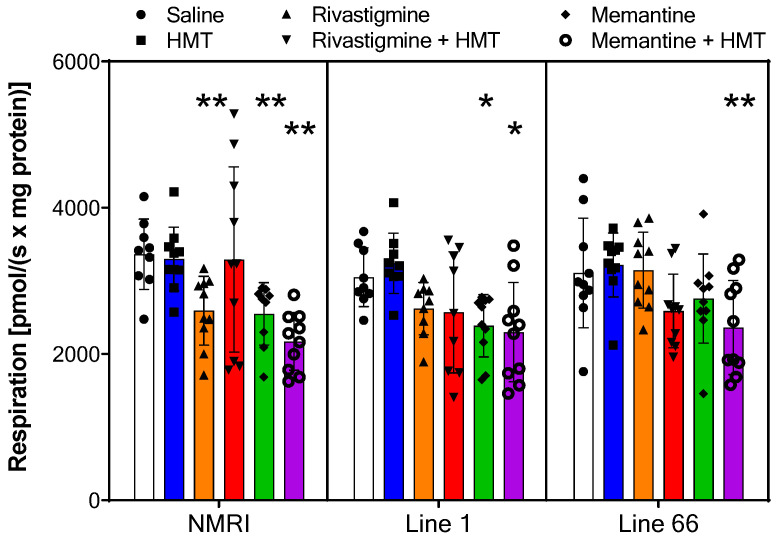
Oxygen flux of *Oxidative Phosphorylation* in NMRI, Line 1 and Line 66 mice with different treatment conditions. Substrates, uncoupler and inhibitors were used to measure oxidative phosphorylation (see Section 2 for details). Raw data were normalized to mitochondrial citrate synthase activity, and data are presented as scatter overlay of bars representing the means ± SD of 10 experiments for each treatment and genotype. Outliers (<50% below mean) were revealed for the following groups: NMRI—saline (n = 1), HMT 1, Rivastigmine + HMT (2), Memantine (1); L1—HMT (1), Rivastigmine (1), Rivastigmine + HMT (1). No significant difference with the term genotype was returned in the two-way ANOVA (F values < 1.5), but there was a reliable difference between treatments (F_5,153_ = 13; *p* < 0.0001). Each genotype was then analyzed by one-way ANOVA followed by Bonferroni’s multiple comparison test and confirmed reductions in oxidative phosphorylation in specific drug cohorts, most robustly when memantine and HMT were co-administered (see asterisks for comparison with saline control: * *p* < 0.05; ** *p* < 0.01).

**Figure 8:**
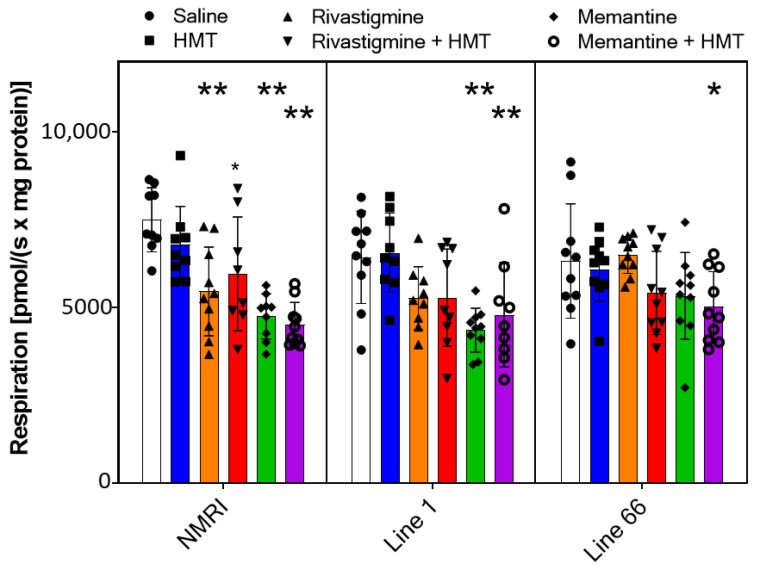
Oxygen flux of *Complex IV* in NMRI, Line 1 and Line 66 mice with different treatment conditions. Substrates, uncoupler and inhibitors were used to measure complex IV (see Section 2 for details). Raw data were normalized to mitochondrial citrate synthase activity, and individual data points are presented as scatter plots and bar chart indicating means ± SD of 10 experiments. Outliers were identified for the following treatment groups: NMRI—saline (n = 1), Memantine (1); L1—HMT (1), Rivastigmine (1), Rivastigmine + HMT (1), Memantine + HMT (1). As for Complex I, II and oxidative phosphorylation before, a two-way ANOVA presented differences in some drug groups (F_5,156_ = 11; *p* < 0.0001), but no other terms. A genotype-selective one-way ANOVA followed by Bonferroni’s multiple comparison test confirmed significant decreases in complex IV activity for specific treatment groups, most prominent in all genotypes for memantine and HMT co-treatment (see asterisks for comparison with saline control: * *p* < 0.05; ** *p* < 0.01).

Pretreatment of the mice with anti-dementia drugs revealed inhibitory properties of rivastigmine and memantine on different complexes of the electron transport chain (Figure 5, Figure 6, Figure 7 and Figure 8). In contrast, no inhibitory action was observed following treatment with HMT alone (Figure 5, Figure 6, Figure 7 and Figure 8). In wild-type mice, rivastigmine decreased oxygen consumption through complexes I, II and IV by 20–25%. Co-treatment with HMT partly prevented this down-regulation, but values did not return to normal. In all genotypes, memantine also reduced activation of all complexes, an effect that was accentuated by co-treatment with HMT (Figure 5, Figure 6, Figure 7 and Figure 8).

In L1 mice, only memantine pre-treatment caused significant inhibition of all mitochondrial complexes whereas the reductions seen after rivastigmine did not reach significance. HMT stimulated oxygen consumption in complexes I and II but did not affect the actions of rivastigmine or memantine (Figure 5, Figure 6, Figure 7 and Figure 8). In L66 mice, memantine and HMT were inactive by themselves but inhibitory in combination. Memantine was slightly inhibitory by itself, but the combination of memantine + HMT caused significant decreases in oxygen consumption (Figure 5, Figure 6, Figure 7 and Figure 8).

### 2.3. HMT Pharmacokinetics

**Figure 9:**
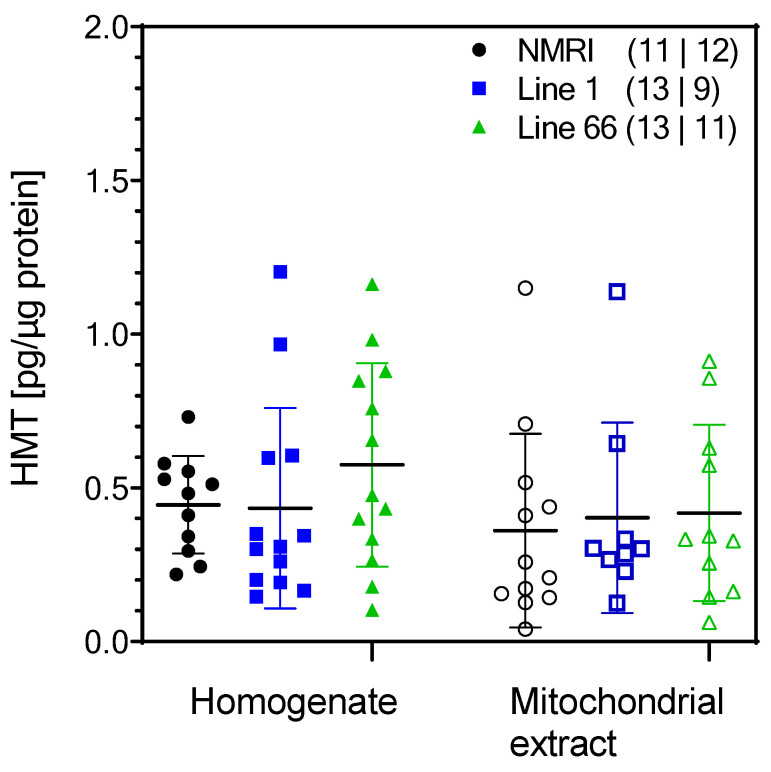
Concentrations of HMT in mouse brain homogenates and in isolated brain mitochondria after administration of HMT for two weeks (5 mg/kg daily). Data represent individual values and group means ± SD of 9–13 experiments (*N* values for homogenates and isolated mitochondria given in brackets). All HMT groups were combined receiving either HMT alone or in combination with rivastigmine or memantine. A two-way ANOVA revealed no differences between genotypes or extracts (F values < 1.6). Note that the level of mitochondrial HMT accumulation is close to levels in the whole homogenate.

We investigated blood and brain levels of HMT after administration of 5 mg/kg by gavage, where the last dose was given 5–6 h prior to sacrifice. Methylthionine (MT) concentrations were determined by HPLC after oxidation of HMT. The plasma concentration of HMT was 12.4 ± 2.2 nM. The HMT concentration in fresh brain homogenate was 0.44 ± 0.05 pg/µg protein. Assuming a protein content of 12% in brain homogenate, this is equivalent to 61.2 ng/g brain tissue, and assuming a brain water content of 80%, this value corresponds to 267 nM in brain water (HMT and MT are water-soluble but highly protein-bound). It follows that HMT is approximately 20-fold more concentrated in the brain than in blood plasma. Moreover, we isolated mitochondria from brain homogenate and measured an HMT concentration of 0.36 ± 0.09 pg/µg protein in mitochondrial suspensions (Figure 9). This value is close to the amount of HMT in brain homogenates (0.44 ± 0.05 pg/µg protein) which demonstrates that HMT distributes freely into mitochondria. The values for HMT in L1 and L66 mice were similar as in wild-type mice (Figure 9).

## 3. Discussion

Mitochondrial dysfunction as a pathological feature of AD has been described in numerous studies (reviewed in [9,12]). Mitochondrial loss and distribution deficits of mitochondria were observed in AD brain [25,26], and mitochondrial fragmentation was observed in hippocampal neurons of AD patients resulting in ATP depletion [27]. Changes of gene expression were documented and concerned both nuclear as well as mitochondria-encoded OxPhos genes [28,29]. Both complex I and complex IV activities were found to be vulnerable in AD patients [30,31,32], e.g., by proteomics [33], and correlated with clinical symptoms [34]. Reductions of mitochondria in presynaptic cortical terminals were described in [35]. Similar changes were found in mouse models of AD, including changes of mitochondrial transport, reduced ATP levels, and increased expression of the mitochondrial fission protein Fis1 [36,37,38]. Of note, mitochondrial dysfunction of aging tau-transgenic mice may explain the cholinergic changes that we have reported previously in tau-transgenic mice [23,39].

In the present work, we focused on energy metabolites and the functional activity of mitochondria by measuring oxygen consumption. Measurement of microdialysates indicated a largely preserved availability of glucose in the brain of wild-type and tau-transgenic mice. However, four weeks of treatment with rivastigmine caused a decrease of glucose levels and an increase in the lactate/pyruvate ratio. These observations are compatible with increased aerobic glycolysis and reduced activity of the citric acid cycle induced by rivastigmine. Neither HMT nor memantine showed similar effects.

The determinations of oxygen flux revealed intact complex I and II activities in tau-transgenic mice but a significant reduction of complex IV activities by 14% in L1 mice and 16% in L66 mice, respectively. Decreased complex IV activity was also found in AD patients [8,40,41] and co-localized with tau pathology [42]. It should be noted that complex IV activity in this study was measured with artificial substrates, and therefore, our activity screen yielded values for complex IV beyond the maximum physiological oxygen consumption which is reflected in the ETS values. In other words, physiological respiration may not be measurably impaired in the tau-transgenic mice because complex IV activity is not rate-limiting for electron transport and ATP formation. Nevertheless, the finding of reduced maximum respiration at complex IV may be indicative of mtDNA injuries that are known to contribute to neurodegeneration [9]. As these defects accumulate with age, it seems possible that older tau-transgenic mice would have shown more prominent impairments of mitochondrial functions, e.g., after ongoing neuroinflammation [43].

The investigation of drug effects on mitochondrial complexes revealed that HMT does not impair mitochondrial activities and may cause minor increases in L1 mice. In contrast, significant reductions of complex I, II and IV activities were observed for rivastigmine and memantine. Treatment with rivastigmine significantly reduced complex I, II and IV activities in NMRI mice, as well as OxPhos and ETS activities. These effects were smaller in L1 mice whereas L66 mice were resistant to rivastigmine-related changes. Treatment with memantine caused reductions of complex activities in all three strains and was particularly strong in combination with HMT. These findings are of major interest because long-term treatment with the established drugs rivastigmine and memantine may decrease mitochondrial respiration in human patients whereas we found no evidence that HMT compromises mitochondrial function. Moreover, this reduction of mitochondrial respiration may partly explain our previous finding that HMT no longer stimulates cholinergic activity when mice were pre-treated with rivastigmine or memantine [23]. The mechanism of action of the drugs remains elusive although memantine (but not rivastigmine) was reported to affect complex I activity even in acute assays [44]. The fact that all complexes show impaired activities under rivastigmine or memantine treatment does not suggest an interaction of these drugs with single complexes but rather points to a change of mitochondrial dynamics. We speculate, therefore, that rivastigmine and (possibly) memantine interfere with mitochondrial fusion and fission processes. It remains elusive whether these interactions may also explain the lower glucose levels observed after rivastigmine administration (Figure 3).

We clearly show that HMT does not influence mitochondrial respiration in vivo. Glucose levels and the lactate/pyruvate ratio were unchanged, and oxygen consumption was unaffected by HMT in our study. This is not caused by insufficient distribution of the drug since plasma and brain levels of HMT and derivatives were measurable and within the range of previous reports [45]. We confirm that brain levels of HMT and its derivatives are several-fold higher than plasma concentrations [46] and report for the first time that HMT concentrations in isolated mitochondria are similar to those in total brain homogenate.

In summary, HMT reaches nanomolar concentrations in the brain and can be found in similar concentrations in isolated brain mitochondria. However, HMT does not affect mitochondrial respiration and even showed some stimulatory effects in L1 mice. In contrast, treatment with rivastigmine and (to some extent) memantine caused reduced oxygen consumption in several complexes, likely due to effects on mitochondrial dynamics.

## 4. Materials and Methods

### 4.1. Animal Model

A truncated core PHF-tau fragment consisting of tau296–390 from the longest human CNS tau isoform htau40 was expressed on an NMRI (Naval Medical Research Institute) background to generate the transgenic line 1 (L1) mouse. The Thy1 regulatory element was chosen to control the expression of this fragment. In the L1 mouse brain, oligomeric tau accumulates, but intracellular neurofibrillary tangles are not observed [47]. Tau protein aggregates occur in hippocampus and entorhinal cortex in L1 mice up to six months of age. By 12–18 months, tau aggregates spread to the neocortex thereby resembling the Braak staging of tau pathology in AD [13]. While cognitive deficits show a disease progression from 3 to 6 months, animals are devoid of sensorimotor anomalies [47]. We here selected 6–8-month-old mice because tau pathology was fully developed at this age and learning deficits were significant [47,48].

The second model, Line 66 (L66) mice, expresses full-length htau40 carrying two point mutations in the repeat domains with pro-aggregating properties: P301S causes FTPD-17-like pathology in mice [49]. G335D promoted tau aggregation in vitro [48]. Expression is also under control of the murine Thy1 promotor. L66 mice present with intraneuronal tau aggregates and filaments which accumulate in the soma and are present in synaptic preparations [50]. In contrast to the region-specific expression pattern seen in L1 mice, L66 mice presented with widespread tau aggregation in subcortical and cortical regions. L66 mice, in contrast to L1 mice, show no deficit in spatial orientation but exhibit a pronounced sensorimotor impairment leading to this being labeled as an FTDP-17 mouse model [43,48].

### 4.2. Animals and Drug Treatment

Results presented in this work were derived from two different study arms: Data denoting levels of energy metabolites in the microdialysate and blood plasma were from the animal cohort for which acetylcholine measurements have been published previously [23]. Data from mitochondrial activity and electron transport chain function were obtained from a separate cohort. Thus, metabolite data were available for NMRI and L1 mice only; data on respiration and mitochondrial activity included all three genotypes, NMRI, L66 and L1. The number of animals per cohort and drug condition is listed in the individual figure legends. Only female mice were used in line with our previous phenotypic descriptions [43,47,48]. Some animals were excluded for various reasons (blood collection failed; microdialysis probe placement incorrect; homogenisation incomplete; recording error due to software or hardware failure of the equipment used). This explains why sample sizes differ for each set of data.

Animals had access to food and water ad libitum and were kept under standard conditions (temperature 20–22 °C, 50–65% relative humidity; 17–20 air changes per hour) and on a 12 h light/dark cycle (07:00 a.m. to 07:00 p.m.). After at least one week of habituation to the animal facility (delivery by air from breeding in positively pressured isolators at Charles River Laboratories, Margate, UK), mice were randomly assigned to study groups (10 per group) by using block randomization (Latin square design). Based upon 5 or 6 animals per cage, mice were assigned to either 5 or 6 groups in the following order: ABCDE, BAECD, CDAEB, DEBAC and ECDBA or ABCDEF, BFDCAE, CDEFBA, DAFECB, ECABFD and FEBADC. All animal procedures were carried out to minimize animal suffering in accordance with German and European law (EU directive 2010/63/EU). Exclusion criteria were death during anesthesia, blocked microdialysis probes, probe leaking or animals under severe pain. Three mice of different genetic background died due to respirational failure during surgery; another nine had to be excluded because of probe leakage. Three mice were tested on day one but were excluded on day two due to blocked probes. The study was registered with the local authorities (RP Darmstadt; FR/1011).

HMT-mesylate (TauRx Therapeutics Ltd., Aberdeen, UK) was stored at 8 °C and administered by oral gavage. The dose is expressed as free base (mg HMT/kg body weight). To minimize oxidation, HMT-mesylate was dissolved in nitrogen-sparged water prior to administration. Rivastigmine (Tocris Bioscience, Bristol, UK; Cat. 129101-54-8) and memantine (Tocris Bioscience, Bristol, UK; Cat. 41100-52-1) were diluted in aqua ad injectabilia prior to filling minipumps (see below).

In Figure 1, the different groups and therapy regimes are displayed. To deliver the drugs constantly over 28 days, rivastigmine (0.5 mg/kg/d) and memantine (1 mg/kg/d) were filled into an osmotic minipump (ALZET^®^ pump model 1004; DURECT, Cupertino, CA, USA) which was implanted four weeks before dialysis and respirometric measurements, respectively. Under isoflurane (Dechra Veterinary Products, Aulendorf, Germany) anesthesia (2.0–2.5%), the filled pump was subcutaneously implanted in the neck region, and the small incisional wound was sealed with wound closure clips (FST, Foster City, CA, USA). The doses were selected according to previous studies in our laboratory [23] and were converted from human doses using a dose conversion routine [51].

Saline (0.2 mL/d) or HMT (5 mg/kg/d) was given daily by oral gavage for two weeks prior to the microdialysis experiment. Drugs and chemicals of general use were supplied by Merck (Darmstadt, Germany) or Sigma-Aldrich (Munich, Germany) at the highest purity available.

### 4.3. Microdialysis

For microdialysis experiments, 60 female NMRI (RRID: IMSR_TAC:nmri) and 60 female L1 mice were housed in groups of 5 or 6 in stock boxes. Mice were single housed in microdialysis cages prior to probe implantation. We used self-built probes [52] that were implanted under general anesthesia and stereotaxic control as previously described [23]. Filtral 12 AN69-HF (Hospal Industrie, Meyzieu, France) was used as microdialysis membrane; it had a molecular cut-off of 10 kDa. The exchange area was limited to 2 mm with silicon glue. Recoveries of glucose and lactate, as determined in vitro, were 8.4 ± 1.1% and 9.0 ± 1.7%, respectively (N = 7). Probes were implanted into the ventral hippocampus under isoflurane anesthesia (coordinates from Bregma: AP—2.7 mm; L—3.0 mm; DV—3.8 mm). A minimum of 18 h was maintained between probe implantation and experimental start.

Microdialysate for metabolite analysis was collected on the experimental day for 90 min. The probes were perfused with artificial cerebrospinal fluid (aCSF): 147 mM NaCl, 2.7 mM KCl, 1.2 mM MgCl_2_ and 1.2 mM CaCl_2_. At the end of the experiment, mice were deeply anesthetized with 5% isoflurane and decapitated, and peripheral blood was collected quickly into an EDTA tube and rapidly centrifuged at 4 °C, 1400 g for 20 min. The supernatant plasma was collected and stored at −80 °C.

### 4.4. Analytical Measurements

Glucose, lactate and pyruvate levels in dialysates and blood plasma were measured by a colorimetric method using an IscusFlex^®^ microdialysis analyzer (M Dialysis AB, Stockholm, Sweden). Samples were thawed on ice, and the IscusFlex was calibrated with its commercially available calibration standards.

### 4.5. Mitochondrial Respirometry

A total of 180 mice (60 NMRI, 60 L1 and 60 L66) were used to obtain mitochondrial data. After four weeks of drug administration (rivastigmine, memantine) and after 14 days of oral gavage (HMT), the animals were sacrificed by decapitation in deep isoflurane anesthesia. The whole brain was harvested. The olfactory bulb and cerebellum were removed. Brain hemispheres weighing 80–120 mg were homogenized in 2 mL ice-cold MiR05 medium supplemented by protease inhibitors, and mitochondria were isolated by a sequence of five centrifugation steps as follows:

1. 1400× *g* for 7 min, then transfer of the supernatant into a new vial for the next centrifugation step;
2. 1400× *g* for 3 min, then transfer of the supernatant into a new vial for the next centrifugation step;
3. 10,000× *g* for 5 min, then the supernatant was discarded, and the pellet suspended with 1 mL MiR05 plus protease inhibitor;
4. 1400× *g* for 3 min, then transfer of the supernatant into a new vial for the final centrifugation step;
5. 10,000× *g* for 5 min, the supernatant was discarded, and the pellet suspended with 1 mL MiR05 plus protease inhibitor to yield the mitochondrial fraction.

An immunoblot of the mitochondrial fraction (R.X. Santos & G. Riedel, Aberdeen) confirmed an enrichment of mitochondrial markers. Mitochondrial oxygen consumption was determined in an Oxygraph-2K (O2K) (Oroboros Instruments GmbH, Innsbruck, Austria) [53]. For this purpose, 80 μL of the mitochondrial suspension was injected into one oxygraph chamber, and the remaining sample was shock-frozen in liquid nitrogen and stored at −80 °C for citrate synthase assays. The MiR05 medium (pH: 7.1) contained 110 mM D-sucrose, 60 mM K^+^ lactobionate, 0.5 mM EGTA, 3 mM MgCl_2_, 20 mM taurine, 10 mM KH_2_PO_4_, 20 mM HEPES and 1 g/L fatty acid-free BSA.

Activities of the complexes I, II and IV, OxPhos activity and electron transfer system (ETS) capacity were determined using an established substrate/uncoupler/inhibitor titration (SUIT) protocol [54,55]. Complex I activity was measured after the addition of pyruvate (5 mM; all chemicals from Sigma-Aldrich, Munich, Germany, P2256), malate (1 mM; Sigma M1000) and ADP (2 mM; Sigma A5285). ATP synthase activity was blocked by the addition of oligomycin (2 µM; Sigma O4876). Maximum electron transfer was measured after the stepwise addition of the protonophore FCCP (12.5 µM; Sigma C2920). Complex II activity was determined after the addition of succinate (10 mM) and inhibition of Complex I by the addition of rotenone (2.5 μM; Sigma R8875). Then, mitochondrial respiration was blocked by the addition of antimycin-A (2.5 µM; Sigma A8674) which inhibits complex III. Subsequently, maximum complex IV activity was measured by the addition of tetramethyl-phenylenediamine (TMPD; 0.5 mM; Sigma A7631) as an electron donor and 2 mM ascorbate (Sigma A7631) to maintain the reduced state of TMPD. Finally, citrate synthase (CS) activity was measured by a colorimetric assay [54], and oxygen consumption was expressed after normalization to CS activities which was used as a quantitative marker of functional mitochondria [56].

### 4.6. Data Analysis and Statistics

This is an exploratory study using metabolite levels and mitochondrial activities as outcomes. No blinding was performed. Normal distribution of data was tested using the Kolmogorov–Smirnov Test. Sample size was calculated by the formula N = 2 SD^2^ × power index/delta^2^. Based on many years of experience, an SD of 20% was expected, and a treatment effect of 25% was defined as the goal for the study. The value for the power index (α = 0.05, two-sided; ß = 0.2; 80%) was taken from *Intuitive Statistics* (Harvey Motulsky, Oxford University Press, 1995). Data were analyzed by one-way Analysis of Variance (ANOVA) with Bonferroni’s post-test. Prism 5.0 (GraphPad Software, San Diego, CA, USA) was used for statistical calculation and plotting. *p*-values less than 0.05 were considered statistically significant. Data are shown as absolute values and given as mean ± SD of *N* experiments.

## Data Availability

All data will be made available on request.

## Abbreviations

AD	Alzheimer’s disease
HMT	hydromethylthionine
LMTM	leucomethylthioninium dihydromesylate
MTC	methylthioninium chloride (methylene blue)
PHF	paired helical filaments
